# Predicting Live Birth, Preterm Delivery, and Low Birth Weight in Infants Born from In Vitro Fertilisation: A Prospective Study of 144,018 Treatment Cycles

**DOI:** 10.1371/journal.pmed.1000386

**Published:** 2011-01-04

**Authors:** Scott M. Nelson, Debbie A. Lawlor

**Affiliations:** 1 Centre for Population and Health Sciences, University of Glasgow, Glasgow, Scotland, United Kingdom; 2 MRC Centre for Causal Analysis in Translational Epidemiology, School of Social and Community Medicine, University of Bristol, Bristol, England, United Kingdom; University of Queensland Centre for Clinical Research, Australia

## Abstract

**Background:**

The extent to which baseline couple characteristics affect the probability of live birth and adverse perinatal outcomes after assisted conception is unknown.

**Methods and Findings:**

We utilised the Human Fertilisation and Embryology Authority database to examine the predictors of live birth in all in vitro fertilisation (IVF) cycles undertaken in the UK between 2003 and 2007 (*n* = 144,018). We examined the potential clinical utility of a validated model that pre-dated the introduction of intracytoplasmic sperm injection (ICSI) as compared to a novel model. For those treatment cycles that resulted in a live singleton birth (*n* = 24,226), we determined the associates of potential risk factors with preterm birth, low birth weight, and macrosomia. The overall rate of at least one live birth was 23.4 per 100 cycles (95% confidence interval [CI] 23.2–23.7). In multivariable models the odds of at least one live birth decreased with increasing maternal age, increasing duration of infertility, a greater number of previously unsuccessful IVF treatments, use of own oocytes, necessity for a second or third treatment cycle, or if it was not unexplained infertility. The association of own versus donor oocyte with reduced odds of live birth strengthened with increasing age of the mother. A previous IVF live birth increased the odds of future success (OR 1.58, 95% CI 1.46–1.71) more than that of a previous spontaneous live birth (OR 1.19, 95% CI 0.99–1.24); *p*-value for difference in estimate <0.001. Use of ICSI increased the odds of live birth, and male causes of infertility were associated with reduced odds of live birth only in couples who had not received ICSI. Prediction of live birth was feasible with moderate discrimination and excellent calibration; calibration was markedly improved in the novel compared to the established model. Preterm birth and low birth weight were increased if oocyte donation was required and ICSI was not used. Risk of macrosomia increased with advancing maternal age and a history of previous live births. Infertility due to cervical problems was associated with increased odds of all three outcomes—preterm birth, low birth weight, and macrosomia.

**Conclusions:**

Pending external validation, our results show that couple- and treatment-specific factors can be used to provide infertile couples with an accurate assessment of whether they have low or high risk of a successful outcome following IVF.

*Please see later in the article for the Editors' Summary*

## Abstract 2

Using the HFEA database of all 144,018 live births in all IVF cycles in the UK between 2003 and 2007, Scott Nelson and Debbie Lawlor show that couple- and treatment-specific factors can be used to help predict successful outcome following IVF.

## Editors' Summary

**Background:**

Worldwide, more than 10% of couples are infertile. Sometimes there is no obvious reason for a couple's inability to have children but, for many couples, problems with their eggs or sperm prevent “fertilization”—the union of an egg and a sperm that leads, eventually, to the birth of a baby. Until recently, little could be done to help infertile couples. Then, on the 25 July 1978, the world's first “test-tube baby” was born. Since then, 4 million babies have been born through in vitro fertilization (IVF). In IVF, mature eggs are collected from the woman (or from an egg donor if the woman cannot make her own eggs) after a course of special hormones, and they are mixed in a dish with her partner's sperm. If her partner has a low sperm count or abnormal sperm, a single sperm can be injected directly into the egg in a procedure called intracytoplasmic sperm injection (ICSI), which became widely available in the mid 1990s, or sperm from a donor can be used. Finally, a number (depending on the country) of embryos (eggs that have begun to divide and develop) are put back into the woman where, hopefully, they will establish a successful pregnancy.

**Why Was This Study Done?:**

Not every attempt at IVF is successful. In the US and the UK, IVF is successful in about a third of women under 35 years old but in only 5%–10% of women over the age of 40. It would be useful to have a way to predict the likelihood of a live birth after IVF for individual couples. Such a “prediction model” would facilitate patient counseling, clinical decision making, and the allocation of IVF resources. In this study, the researchers use information on IVF cycles collected by the Human Fertilisation and Embryology Authority (HFEA), which regulates IVF in the UK, to assess the extent to which the characteristics of infertile couples and the treatment they receive can be used to predict live birth after IVF. They also use these data to identify which factors are associated with preterm delivery, low birthweight, and macrosomia (the birth of an unusually large baby), three undesirable birth characteristics.

**What Did the Researchers Do and Find?:**

Between 2003 and 2007, 163,425 IVF cycles were completed in the UK, 23.4% of which resulted in at least one live birth. The researchers used the data collected by the HFEA on 144,018 of these cycles (the other cycles had missing data) to develop a multivariable logistic regression prediction model (a type of statistical model) for the outcome of IVF. According to this model, a decreased chance of at least one live birth was associated with several factors including increasing maternal age, increasing duration of infertility, and the use of the woman's own oocytes. By contrast, a previous IVF live birth and the use of ICSI were associated with increased chances of success. Importantly, compared with an established multivariable prediction model, which was developed before the introduction of ICSI, the researchers' new prediction model predicted the chance of a live birth following IVF with greater accuracy. Finally, the researchers report that the chances of preterm and low birthweight after IVF were increased if donor eggs were required and ICSI was not used, that an increased risk of macrosomia was associated with increasing maternal age and with a history of previous live births, and that all three undesirable birth characteristics were associated with infertility due to cervical problems.

**What Do These Findings Mean?:**

These findings indicate that couple- and treatment-specific factors can be used to provide infertile couples with an accurate assessment of whether they have a low or high chance of a successful outcome following IVF. The prediction model developed here provides a more accurate assessment of likely outcomes after IVF than a previously established model. Furthermore, because the new model considers the effect of ICSI on outcomes, it should be more useful in contemporary populations than the established model, which does not consider ICSI. However, before this new prediction model is used to guide clinical decisions and to counsel patients, it needs to be validated using independent IVF data. To facilitate the external validation of their model, the researchers are currently generating a free web-based prediction tool and iPhone application (IVFpredict).

**Additional Information:**

Please access these websites via the online version of this summary at http://dx.doi.org/10.1371/journal.pmed.1000386.

- The Human Fertilisation and Embryology Authority provides information on IVF and IVF statistics for the UK
- The UK National Health Service Choices website provides information for patients on infertility and on IVF
- The American Pregnancy Association has information for patients on infertility and on IVF
- MedlinePlus has links to further resources on infertility and IVF (in English and Spanish)
- The history of the development of IVF is described on the Nobel Prize website
- The prediction tool that was used in this study is at http://www.IVFpredict.com

## Introduction

In-vitro fertilisation (IVF) is now widely used for the treatment of infertility, and validated age-stratified national success rates and outcomes are published annually [1],[2],[3]. To facilitate patient counselling, clinical decision-making, and access to health care provision, prediction models for live birth after IVF have been constructed [4]. However, these studies have been limited by their sample size, development before the introduction of intracytoplasmic sperm injection (ICSI), or lack of validation in external populations [5],[6],[7],[8],[9]. Established multivariable prediction models may therefore not be applicable to contemporary couples seeking treatment. Consequently, clinicians and regulatory bodies have not adopted prediction models and predominantly quote age-related success rates [1],[2],[3].

Given the known complications with multiple gestations and prematurity, the focus has moved to defining the most appropriate IVF outcome variable as a singleton term live birth [10],[11],[12]. Low birth weight and macrosomia are also known to be associated with immediate and long-term risk to offspring heath [13], and IVF singletons are at increased risk of these complications [14],[15]. It is now recognised that factors leading to infertility may be responsible for adverse perinatal outcome rather than the process itself [16],[17],[18],[19]; however, which parental characteristics of infertile couples contribute to adverse perinatal outcomes in IVF singletons and can thereby be targeted for intervention remain unknown.

In this prospective cohort study of 144,018 treatment cycles we assessed the extent to which baseline characteristics can be used to predict live birth after IVF-assisted conception, and for those cycles in which a singleton pregnancy was achieved we identified which factors were associated with preterm delivery, low birth weight, and macrosomia.

## Methods

### Source of Data

**Figure 1:**
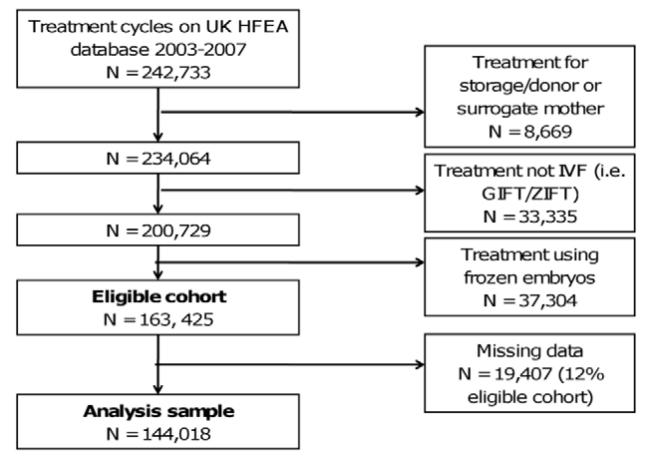
Definition of eligible cohort and analysis sample. IVF, In-vitro fertilisation, GIFT, gamete intra-fallopian tube transfer, ZIFT, zygote intra-fallopian tube transfer.

The UK Human Fertilisation and Embryology Authority (HFEA), which is responsible for the regulation of assisted conception treatment in the UK, has had a Parliamentary statutory obligation to prospectively collect baseline information and birth outcomes on all licensed fertility treatment cycles performed in the UK since 1991 [7]. All treatment cycles and outcomes registered on the HFEA database between January 2003 and December 2007 were used in our study, with the final analysis cohort details and exclusion criteria provided in Figure 1 [20]. Treatment cycles that were for storage or donation of gametes, were not IVF, or were frozen embryo transfers were excluded. Although there is a move to greater use of frozen embryo cycles we excluded these from our analyses to be consistent with previous publications, including that by Templeton et al. [7] in which the established model was developed. Furthermore, during the time studied very few elective single embryo transfers were performed (<0.05% of all cycles). HFEA data relating to treatments between April 1999 and March 2002 were not verified by licensed treatment centres and are therefore deemed less accurate. Furthermore, few treatment cycles had treatment with ICSI before and during this period. Whilst data have been collected beyond 2007, validation checks on the computerised data undertaken by HFEA are currently complete only to December 2007. Anonymised data were provided by the HFEA per cycle of treatment rather than for individual women, so our outcomes are expressed as rates and/or odds per cycle of treatment (rather than per individual woman). Ethical approval of the study was provided by the HFEA.

### Measurements

Maternal age, duration and cause of infertility, previous number of IVF attempts, number of previous spontaneous and IVF live births, source of gametes, and cycle number were recorded at the time of treatment. Duration of fertility, number of previous IVF attempts, number of previous pregnancies, number of previous IVF pregnancies, and total number of previous live births were all categorised in accordance with the previous analysis by Templeton [7]. Cycle number was collapsed, with more than three cycles as one category, because of small numbers. Live birth was defined as a baby born alive after 24 wk gestation. Our main outcome was at least one live birth, which was defined as any birth event in which at least one baby was born alive and survived for more than 1 mo. This outcome is consistent with previous publications, including that by Templeton et al. [7] used to define the established prediction model. In a sensitivity analysis we repeated associations with this main outcome after exclusion of multiple pregnancies, defined as those in which two or more fetal heartbeats were noted at 8 wk gestation.

For assessment of perinatal outcomes in cycles with a singleton live birth, gestational age at birth was defined as completed weeks of gestation. For the main outcome of preterm we examined multivariable associations with preterm birth, defined as ≤36 completed weeks; we also examined associations with extreme preterm (<33 wk). Birth-weight outcomes were supplied in 500 g increments and categorised as low birth weight (LBW <2.5 kg), normal (≥2.5 to <4.0 kg) or macrosomic (≥4 kg). In these analyses we included only cycles in which there was one heartbeat at 8 wk gestation and one live birth (i.e. these were singleton live births).

### Statistical Methods

We performed univariable and multivariable logistic regression to assess associations with at least one live birth. Given that the historical multivariable Templeton model [7] had been externally validated we first tested its predictive ability. We used the reported characteristics that were associated with live birth and their respective regression coefficients from that model to generate the probability of live birth in our cohort (see Text S1 for full details of these calculations) [7]. The predictive ability of the model was assessed by determining the discrimination, using the area under the curve of receiver operator characteristics (AUROC), and its calibration. Calibration was assessed by ranking participants into tenths based on their predicted risk for the Templeton prediction model, and then within each tenth comparing the predicted mean rate to the observed rate of live birth.

The multivariable logistic regression model formed the basis of our novel prediction model of live birth. In the novel prediction model we used the same characteristics as those used in the Templeton model but included all causes of infertility (Templeton includes only tubal versus all other causes), and allowed the coefficients for this and all other variables to be newly derived, and included four additional characteristics—the source of the egg (donor or patient's own), type of hormonal preparation used (antioestrogen, gonadotrophin, or hormone replacement therapy), whether or not ICSI was used, and the number of the treatment cycle (1, 2 or ≥3). We tested all two-way interactions between pairs of predictors included in our multivariable analyses and used a Bonferroni-corrected (for multiple testing) *p*-value threshold of 0.05 to define statistical evidence of an interaction. The discrimination and calibration of this novel model was assessed as described above. The AUROC between the Templeton model and our model was compared using the ROCCOMP command in Stata [21]. When we repeated the multivariable analyses using 1,000 bootstrap replications, the estimates and their standard errors were essentially the same and results are presented here without bootstrapping.

Lastly, we examined model reclassification by determining the integrated discrimination improvement (IDI) of the novel prediction model compared to the original Templeton model [7]. The IDI is a summary measure of the extent to which a new prediction model increases risk prediction in individuals who ultimately have the outcome of interest [22], and reduces risk prediction in those who remain healthy in comparison to the established risk prediction model (in this case the Templeton model [7]).

To explore risk factors for adverse perinatal outcomes (preterm, extreme preterm, low birth weight, and macrosomia) we used logistic regression to examine the univariable and independent multivariable associations of all risk factors assessed in the multivariable analyses of at least one live birth, as described above. The selection of these potential risk factors for adverse perinatal outcomes was based on previous studies and the plausibility that risk factors that influence odds of live birth are also likely to affect gestational age and birth weight. For associations with preterm as the outcome we additionally adjusted for mean birth weight, and for outcomes with low birth weight and macrosomia we adjusted for mean gestational age. These analyses were conducted only for cycles in which there was only one heartbeat at 8 wk gestation and at least one live birth.

All statistical analyses were performed using Stata version 11 (StataCorp LP).

### Dealing with Missing Data

For the vast majority of variables there was no missing data; 3.9% of cycles had missing data on method of hormonal preparation used and 8.4% had missing data on duration of infertility; overall 12% of the eligible cohort had some missing data (Figure 1 and Table S1). Univariable associations were very similar when maximum numbers for each variable were used (Table S2) and when only those with complete data were used (Table 1), suggesting that missing data did not result in bias.

**Table 1:** Associations of potential predictors for live birth following IVF.

Characteristic	Categories	Univariable Odds Ratio of Live Birth (95% CI)	Multivariable^a^ Odds Ratio of Live Birth (95% CI)	*p*-Value^b^
Maternal age (years)	18–34	1	1	<0.001
	35–37	0.77 (0.75–0.79)	0.78 (0.76–0.81)	
	38–39	0.53 (0.51–0.55)	0.53 (0.51–0.56)	
	40–42	0.29 (0.28–0.30)	0.29 (0.28–0.31)	
	43–44	0.10 (0.09–0.12)	0.10 (0.09–0.12)	
	45–50	0.15 (0.12–0.19)	0.12 (0.09–0.15)	
Duration of infertility (years)	<1	1.48 (1.34–1.65)	1.51 (1.35–1.68)	<0.001
	1–3	1.10 (1.07–1.13)	1.11 (1.08–1.15)	
	4–6	1	1	
	7–9	0.91 (0.87–0.94)	0.94 (0.91–0.98)	
	9–12	0.81 (0.76–0.85)	0.87 (0.82–0.92)	
	>12	0.71 (0.67–0.75)	0.89 (0.84–0.95)	
Cause of infertility	Unknown	1	1	<0.001
	Tubal only	0.94 (0.90–0.97)	0.87 (0.83–0.90)	
	Anovulatory only	0.93 (0.88–0.98)	0.95 (0.90–1.00)	
	Endometriosis only	1.05 (0.98–1.13)	0.96 (0.89–1.03)	
	Cervical only	0.41 (0.20–0.85)	0.39 (0.19–0.82)	
	Male only	1.16 (1.13–1.20)	0.91 (0.87–0.95)	
	Combination known causes	1.01 (0.96–1.06)	0.88 (0.83–0.92)	
Number of previous unsuccessful IVF	0	1	1	<0.001
	1	0.74 (0.70–0.79)	0.72 (0.65–0.81)	
	2	0.69 (0.64–0.76)	0.70 (0.62–0.80)	
	3	0.74 (0.66–0.84)	0.77 (0.66–0.91)	
	4	0.51 (0.42–0.62)	0.55 (0.45–0.69)	
	≥5	0.57 (0.48–0.69)	0.68 (0.55–0.83)	
Mutually exclusive categories of previous IVF and obstetric history	No previous IVF, 0 pregnancy	1	1	<0.001
	No previous IVF, at least 1 pregnancy, 0 live births	0.88 (0.86–0.91)	1.03 (0.99–1.06)	
	No previous IVF, at least 1 pregnancy, at least 1 live birth	0.92 (0.88–0.96)	1.19 (1.14–1.24)	
	Previous IVF, 0 pregnancy	0.72 (0.68–0.76)	1.14 (1.01–1.28)	
	Previous IVF, at least 1 pregnancy, 0 live birth	0.68 (0.64–0.73)	1.02 (0.93–1.11)	
	Previous IVF, at least 1 pregnancy, at least 1 live birth	1.10 (1.03–1.17)	1.58 (1.46–1.71)	
Hormonal preparation	Antioestrogen	1	1	<0.001
	Gonadatrophin	1.43 (1.24–1.63)	1.33 (1.15–1.53)	
	Hormone replacement	1.61 (1.38–1.89)	1.55 (1.31–1.82)	
Cycle number	1	1	1	<0.001
	2	0.80 (0.78–0.83)	0.85 (0.82–0.87)	
	≥3	0.76 (0.74–0.79)	0.88 (0.85–0.91)	
Source of egg	Donor	1	1	<0.001
	Patient	0.87 (0.74–1.02)	0.38 (0.32–0.45)	
Treatment type	IVF	1	1	<0.001
	ICSI plus IVF	1.28 (1.25–1.31)	1.27 (1.23–1.31)	

*N* = 144,018 analysis cohort with complete data on all variables included in any model.

a Multivariable adjusted  =  mutual adjustment for all variables listed in column one.

b *p*-Value for multivariable association; all *p*-values are likelihood ratio tests of null hypothesis that the odds are the same for each category (i.e., they do not assume linearity).

## Results

Figure 1 shows how we established the eligible cohort of IVF treatment cycles (*N* = 163,425) and the sample used for the main multivariable analyses (i.e. without any missing data *N* = 144,018; 88% of eligible). Table S1 shows the study characteristics. Amongst the 163,425 eligible cohort, the overall rate of at least one live birth was 23.4 per 100 cycles (95% CI 23.2–23.7). Rates of successful live birth increased linearly over time from 22.7 per 100 cycles in 2003 to 24.9 per 100 cycles in 2007 (*p*<0.001 for linear trend) (Figure S1).

Table 1 shows univariable and multivariable associations of live birth. The odds of successful live birth decreased with increasing maternal age, increasing duration of infertility, greater number of previously unsuccessful IVF treatments, when the woman's own egg (as opposed to donor) was used, and when this was the second or third (as opposed to first) treatment cycle. Odds of successful live birth were lower when the cause of infertility was tubal, anovulatory, or cervical disease or when it was due to a male cause. Women who had at least one previous live birth (either natural or with IVF) had increased odds of a successful live birth with this cycle, as did those in whom gonadotrophin or hormone replacement (as opposed to antioestrogens) were used and ICSI was used with IVF. A previous IVF live birth increased the odds of future success (OR 1.58, 95% CI 1.46–1.71) more than previous spontaneous live birth (OR 1.19, 95% CI 0.99–1.24); *p*-value for difference in estimate <0.001 (estimated using 1,000 bootstrap replications to estimate standard errors of differences between the log odds between the two regression coefficients).

There was statistical evidence for four interactions, and stratified analyses reflecting these interactions are shown in Table S3 (for interactions with age) and Table S4 (for interactions with ICSI). The increased odds of success in cycles in which the duration of infertility was less than one year increased with increasing maternal age, though only a very small proportion of all cycles were in the category of less than one year duration of infertility. The reduced odds of successful outcome amongst own versus donor oocytes strengthened with increasing age. In couples who had not used ICSI all three causes—male infertility, infertility due to cervical disorders, and infertility due to a combination of causes—were associated with reduced odds of live birth, whereas there were no such associations in those using ICSI. Requiring three or more treatment cycles was associated with reduced odds of live birth in those in which ICSI was used, but not where it was not used. These four interactions were included in our novel prediction model, which is described in Text S2.

**Figure 2:**
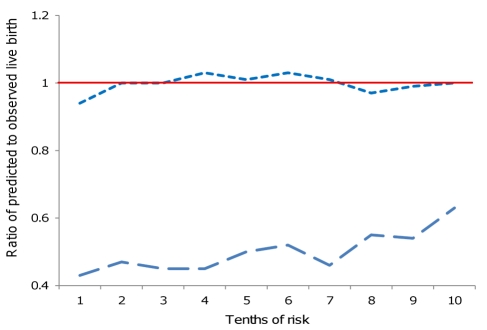
Ratios of predicted to observed live birth rate using two prediction models. *N* = 144,018 cycles of IVF treatment in the United Kingdom. Long dashed line, Templeton model; short dashed line, novel prediction model 2; red horizontal line, ratio of 1 (i.e., perfect prediction) for all levels of risk.

Table 2 shows the AUROC for each of the Templeton and our new prediction models, with statistically significant improvement in discrimination for our novel model. Figure 2 and Table 3 show the observed to predicted rate of successful live birth by tenths of the distribution of the linear prediction models for each of the models. Calibration was poor with the Templeton model, which markedly underestimated the likelihood of successful live birth across the entire distribution of risk, particularly in those at lowest risk. By contrast the novel model had excellent calibration and reclassified cycle probability of a live birth in a way that improved upon the original Templeton model (IDI = 2.1%, *p*<0.001 comparing the novel model to the Templeton [7]).

**Table 2:** AUROC for the Templeton and novel method of predicting live birth with IVF.

Model	AUROC (95% Confidence Interval)	*p*-Value Comparing Models
Templeton	0.6184 (0.6152–0.6217)	ref
Novel model	0.6335 (0.6202–0.6367)	<0.001

*N* = 144,018 analysis cohort with complete data on all variables included in any model. Templeton: As in reference [7], to date the only externally validated prediction model. Novel model: using the same variables as Templeton but allowing them to have different multivariable coefficients to those originally derived by Templeton and including terms for all causes of infertility (rather than just tubal versus other, as in the original Templeton) and four additional predictors: type of hormonal preparation, whether egg came from patient or donor, number of treatment cycles, and whether ICSI was used with the IVF.

**Table 3:** Calibration of the two prediction models.

Tenth of the Distribution of the Linear Predictor	Templeton Prediction Model^a^			Novel Prediction Model^b^		
	Observed Live Birth Rate per 100 Cycles of Treatment (95% CI)	Predicted Live Birth Rate per 100 Cycles of Treatment (95% CI)	Ratio Predicted to Observed	Observed Live Birth Rate per 100 Cycles of Treatment (95%CI)	Predicted Live Birth Rate per 100 Cycles of Treatment (95%CI)	Ratio Predicted to Observed
Lowest 10^th^	8.36(7.91–8.81)	3.57(3.54–3.59)	0.43	7.66(7.23–8.09)	7.23(7.18–7.28)	0.94
2^nd^	13.66(13.10–14.22)	6.44(6.43–6.45)	0.47	13.21(12.66–13.76)	13.16(13.13–13.18)	1.00
3^rd^	18.67(18.04–19.29)	8.45(8.43–8.46)	0.45	18.15(17.53–18.78)	18.00(17.99–18.02)	1.00
4^th^	22.77(22.14–23.40)	10.26(10.25–10.26)	0.45	20.42(19.76–21.08)	21.10(21.09–21.11)	1.03
5^th^	23.30(22.51–24.07)	11.61(11.60–11.62)	0.50	23.40(22.71–24.08)	23.63(23.62–23.63)	1.01
6^th^	25.41(24.77–26.05)	13.31(13.30–13.32)	0.52	24.87(24.18–25.56)	25.57(25.57–25.59)	1.03
7^th^	29.93(29.22–30.64)	13.69(13.69–13.69)	0.46	27.04(26.30–27.77)	27.43(27.42–27.44)	1.01
8^th^	26.78(25.87–27.67)	14.77(14.76–14.78)	0.55	30.37(29.62–31.12)	29.35(29.34–29.36)	0.97
9^th^	31.85(31.21–32.50)	17.29(17.28–17.30)	0.54	32.26(31.54–32.98)	31.95(31.94–31.97)	0.99
Highest 10^th^	33.26(32.23–34.22)	20.91(20.84–20.98)	0.63	36.50(35.66–37.34)	36.47(36.43–35.61)	1.00

*N* = 144,018 analysis cohort with complete data on all variables included in any model. The cohort is split into 10ths of the distribution of the linear predictor for each of the two prediction models. For example, for the Templeton prediction model the observed and predicted are compared by 10^th^ of the Templeton linear predictor.

a Templeton: As in reference [7], to date the only externally validated prediction model (Text S1).

b Novel model: Using the same variables as Templeton but allowing them to have different multivariable coefficients to those originally derived by Templeton and including terms for all causes of infertility (rather than just tubal versus other as in the original Templeton) and four additional predictors: type of hormonal preparation, whether egg came from patient or donor, number of treatment cycles, and whether ICSI was used with the IVF (Text S2).

Of the 144,018 cycles 9931 (7%) were multiple pregnancies (i.e. had two or more fetal heart beats noted at 8 wk gestation). Of these, 1,264 (13%) resulted in one live birth, 7,925 (80%) in two live births, and 109 (1%) in three live births; 633 (6%) did not result in a live birth. When we removed these 9,931 cycles from our analyses results were essentially unchanged from those presented here. For example, Table S5 shows the univariable and multivariable associations of potential predictors with live birth after these exclusions (i.e., the equivalent of Table 1 in this paper). The AUROC, observed to predicted ratios and IDI were the same as those presented in Tables 2 and 3 with these exclusions.

Table 4 provides examples of how our novel prediction model could be used in clinical practice to give an estimate of a couple's probability of achieving a live birth in a given cycle of treatment. This illustrates not only the clinical use of this model (which we have developed into a freely available computer programme, http://www.IVFpredict.com, and iPhone/Android application, IVFpredict) but also how both couple characteristics and treatment choice influence prognosis.

**Table 4:** Examples of risk prediction in women.

Example Couples	Estimate of Probability of Live Birth after IVF per 100 Cycles
A. The woman is 40 y old and the couple have been trying to conceive for over 11 y. They have had four previous unsuccessful IVF treatments (two of which resulted in pregnancy but not a live birth). The couple's cause of infertility is a male problem and they have been treated with ICSI. They are now wishing to embark on their fifth treatment cycle. The woman's own oocytes will be used and the hormonal preparation is gonadotrophin.	4.8/100 cycles
B. If we take the same couple as in A but change the treatments so that a donor oocyte is used and the hormonal preparation is hormone replacement (all other characteristics stay the same as in A)	16.7/100 cycles
C. The woman is 33 and the couple started trying to conceive 5 y ago after the live birth of their son, which was a spontaneous pregnancy. The couple's cause of infertility is unknown. They will not be treated with ICSI; the woman's own oocytes will be used and the hormonal preparation will be gonadotrophins. This will be their first treatment cycle.	29.8/100 cycles
D. If we take the same couple as in C but change the treatments so that ICSI will be used, with a donor oocyte and hormone replacement as the hormonal preparation (all other characteristics stay the same as in A).	43.5/100 cycles

These examples are plausible in terms of the types of patients regularly seen in IVF clinics, and they show the influence of couple characteristics (compare A to C and B to D) and of treatment effects (compare B to A and D to C), and of both of these combined (compare D to A).

Of the 144,018 cycles included in our main analyses for prediction of successful live birth, there were 24,226 live singleton births; 24,096 (99.5%) of these had gestational age data and 24,050 (99.3%) had birth weight data. Mean (SD) gestational age was 38.98 (2.12) completed weeks, with 472 (2.0%) being less than 33 wk, 1,598 (6.6%) between 33–36 weeks and 22,026 (91.4%) 37 or greater weeks. Mean (SD) birth weight was 3.277 (0.629) kg, with 2,100 (8.7%) low birth weight (<2.5 kg), 19,704 (81.9%) a healthy birth weight, and 2,246 (9.3%) macrosomic (≥4.0 kg). Table S6 shows the univariable associations of risk factors preterm (<37 completed weeks), low birth weight (<2.5 kg) and macrosomia (≥4.0 kg). Table 5 shows the multivariable associations of these risk factors with preterm birth, low birth weight, and macrosomia. In multivariable analyses the odds of both preterm birth and low birth weight in singleton IVF live births were reduced when the woman's (rather than donor) egg was used and when ICSI was used. The odds of low birth weight were also reduced with increasing maternal age and with a history of previous pregnancy (either spontaneous or following IVF). Odds of macrosomia increased with increasing maternal age and in cycles in which there was history of a previous pregnancy (either spontaneous or IVF). Odds of all three factors—preterm birth, low birth weight, and macrosomia—were increased when infertility was due to a cervical disorder. Tubal causes of infertility were associated with increased odds of preterm birth, anovulatory causes with low birth weight, and male causes of infertility with macrosomia. Table S7 shows univariable and multivariable associations with extreme preterm birth (<33 wk; *n* = 472). Characteristics that were associated with preterm birth in general were also associated with extreme preterm birth. In addition, extreme preterm birth was lower in 38- to 39-year-olds compared to all other ages and was increased in those who had a previous history of IVF.

**Table 5:** Multivariable associations of potential risk factors with preterm birth, low birth weight and macrosomia amongst singleton births following IVF.

Characteristic	Categories	Multivariable Association with Preterm Birth; *N* = 24,096 in Analyses with *n* = 2,070 Cases of Preterm Birth		Multivariable Association with Low Birth Weight; *N* = 21,804 in Analyses with *n* = 2,100 Cases of Low Birth Weight		Multivariable Association with Low Birth Weight; *N* = 21,950 in Analyses with *n* = 2,246 Cases of Macrosomia	
		Odds Ratio (95%CI)	*p*-Value	Odds Ratio (95%CI)	*p*-Value	Odds Ratio (95%CI)	*p*-Value
Age, y	18–34	1	0.12	1	0.04	1	0.01
	35–37	0.90 (0.80–1.00)		0.87 (0.78–0.97)		1.14 (1.03–1.27)	
	38–39	0.87 (0.75–1.00)		0.86 (0.74–0.99)		1.09 (0.95–1.25)	
	≥40	0.93 (0.77–1.10)		0.96 (0.80–1.15)		1.25 (1.06–1.48)	
Duration of infertility, y	<1	1.05 (0.73–1.49)	0.004	0.86 (0.58–1.25)	0.01	0.81 (0.56–1.17)	0.20
	1–3	0.96 (0.86–1.07)		0.90 (0.80–1.00)		0.90 (0.81–0.99)	
	4–6	1		1		1	
	7–9	1.17 (1.02–1.35)		1.07 (0.93–1.24)		1.02 (0.89–1.16)	
	≥9	1.25 (1.07–1.46)		1.16 (1.00–1.36)		0.93 (0.80–1.09)	
Cause	Unknown	1	<0.001	1	0.02	1	0.002
	Tubal only	1.22 (1.06–1.41)		1.12 (0.97–1.30)		1.06 (0.91–1.22)	
	Anovulatory only	1.14 (0.95–1.37)		1.19 (1.00–1.42		1.04 (0.86–1.25)	
	Endometriosis only	0.86 (0.65–1.14)		1.04 (0.80–1.35)		0.96 (0.73–1.25)	
	Cervical only	9.09 (2.01–41.13)		15.62 (2.59–94.06)		10.46 (1.46–74.85)	
	Male only	0.95 (0.83–1.09)		1.02 (0.89–1.17)		1.22 (1.07–1.39)	
	Combination known causes	1.19 (1.00–1.41)		1.15 (0.97–1.36)		0.92 (0.77–1.11)	
Previous unsuccessful IVF, number	0	1	0.44	1	0.37	1	0.95
	1	0.75 (0.50–1.15)		0.85 (0.56–1.29)		0.92 (0.77–1.10)	
	2	0.80 (0.50–1.30)		0.82 (0.51–1.32)		0.92 (0.61–1.38)	
	≥3	0.66 (0.40–1.11)		0.63 (0.38–1.06)		0.98 (0.61–1.58)	
Mutually exclusive categories of previous IVF and obstetric history	No previous IVF, 0 pregnancy	1	0.11	1	0.004	1	<0.001
	No previous IVF, at least 1 pregnancy, 0 live births	1.15 (0.40–1.11)		1.13 (1.00–1.27)		1.23 (1.09–1.39)	
	No previous IVF, at least 1 pregnancy, at least 1 live birth	0.99 (0.84–1.16)		0.83 (0.70–0.98)		1.28 (1.11–1.49)	
	Previous IVF, 0 pregnancy	1.48 (0.95–2.30)		1.54 (1.00–2.39)		0.97 (0.63–1.49)	
	Previous IVF, at least 1 pregnancy, 0 live birth	1.30 (0.93–1.80)		1.37 (0.99–1.91)		1.02 (0.72–1.43)	
	Previous IVF, at least 1 pregnancy, at least 1 live birth	1.02 (0.55–1.38)		0.89 (0.67–1.17)		1.30 (1.03–1.63)	
Hormonal preparation	Antioestrogen	1	0.11	1	0.77	1	0.86
	Gonadatrophin	0.87 (0.55–1.39)		1.18 (0.71–1.97)		1.08 (0.64–1.83)	
	Hormone replacement	1.07 (0.63–1.82)		1.24 (0.69–2.21)		1.15 (0.64–2.07)	
Cycle number	1	1	0.43	1	0.17	1	0.04
	2	1.00 (0.89–1.13)		0.96 (0.85–1.08)		1.07 (0.96–1.20)	
	≥3	0.92 (0.81–1.05)		0.89 (0.78–1.01)		1.17 (1.04–1.31)	
Source of egg	Donor	1	<0.001	1	<0.001	1	0.16
	Patient	0.41 (0.26–0.64)		0.42 (0.26–0.68)		0.68 (0.39–1.17)	
Treatment type	IVF	1	0.05	1	0.05	1	0.46
	IVF and ICSI	0.89 (0.80–1.00)		0.89 (0.80–1.00)		0.96 (0.86–1.07)	

Cycles included in analyses are from couples for whom data were complete on all variables and who experienced a singleton birth after IVF. For associations with low birth weight, those with macrosomia as an outcome are removed so that low birth weight is compared with normal birth weight, and similarly for macrosomia, those with low birth weight are removed so that macrosomia is compared with normal birth weight. The results are with mutual adjustment for all variables in the first column. In addition, for preterm birth, results are adjusted for mean birth weight and for low birth weight and macrosomia for mean gestational age. *p*-Values are likelihood ratio tests of null hypothesis that the odds are the same for each category (i.e., they do not assume linearity).

## Discussion

In this study we identify precise estimates of the strength and independence of the factors affecting the odds of IVF success and their association with adverse perinatal outcome. To date, successful prediction of live birth after assisted conception has been limited, with a recent systematic review [4] finding that models were limited by their sample size, incorporating fewer than 3,100 cycles or couples and their lack of external validation. The notable exception was the model of Templeton et al., which analysed 36,961 treatment cycles undertaken in the UK between 1991 and 1994 and was validated in a population of 1,253 couples receiving IVF treatment in The Netherlands between 1991 and 1999 [7],[23]. Since then, ICSI for male factor infertility has been widely adopted, and consequently we demonstrate that this previously validated model, although showing reasonable discrimination, is poorly calibrated and of limited use in contemporary populations. We have developed a new model, which encompasses a series of new measures including use of donor oocytes, ICSI, cycle number, and whether there had been a previous spontaneous or IVF-related live birth or fetal loss. Using this novel model we can statistically significantly improve the overall prediction of live birth as assessed by area under the curve and attain excellent calibration with accurate identification of couples with a poor, moderate, or good prognosis. We also find that maternal characteristics, in particular maternal age, source of the oocyte and cervical causes of infertility are strongly associated with the risk of low birth weight and preterm delivery in singleton live births resulting from IVF. Notably, some of these associations were in the opposite direction to those seen for successful live birth. Thus, in women who successfully have a singleton live birth with IVF, the risk of low birth weight is reduced in older compared with younger women and both low birth weight and preterm are reduced when the woman's own embryo has been used.

The use of assisted conception has increased dramatically over recent years, with concomitant increases in success rates, in part driven by the widespread uptake of ICSI for male factor infertility [24]. The importance of ICSI in general and in particular causes of infertility is demonstrated in our study by its association with increased odds of successful live birth and by the fact that couples with male causes of infertility, cervical causes, or combined causes have reduced odds of success if ICSI has not been used, but are unrelated to success if ICSI has been used. Recent technical advances have, however, failed to overcome the reduction in success rates associated with increasing duration of infertility, necessity for repeated IVF attempts, or increasing maternal age, all of which are independently associated with reduced odds of live birth. The detrimental impact of prolonged infertility suggests that early recourse to treatment is appropriate and that extended treatment waiting times, for example whilst trying lifestyle interventions, might militate against eventual success. The marked reduction in the success of the second cycle but then a relative plateau is in contrast to previous reports, which suggested a subtle decline with increasing cycle number [7]. This difference may indicate that previous declines in success rates with increasing cycle number principally reflected increasing maternal age, which we have adjusted for.

In keeping with all previous reports, live birth rates decline with increasing maternal age [2],[4],[7],[23]. By contrast, ours is the first study that we are aware of to find that, in women with successful IVF delivery of a singleton live birth, younger maternal age is associated with increased risk of low birth weight. This latter finding is however, in keeping with the recent observation that maternal age is positively associated with first trimester growth [25], which if impaired is an important determinant of later adverse perinatal outcome [26],[27]. For older women, the use of donor oocytes is a successful strategy for the attainment of a live birth, however, we identify that donor oocyte recipients have a marked increase in the risk of delivering a preterm or low birth weight infant. This may reflect the primary relationship between ovarian senescence and vascular function. Premature and natural menopause have both been associated with widespread vascular dysfunction, dyslipidaemia, a proinflammatory phenotype, and an increased risk of cardiovascular events [28],[29],[30]. These same factors have been implicated in the aetiology of fetal growth restriction and preeclampsia, the major determinants of preterm birth [31]. Furthermore, increased incidence of these complications have been reported in young and old donated oocyte recipients [32],[33]. With respect to macrosomia the associations with older maternal age may reflect higher maternal socioeconomic class due to deferred child bearing or increased maternal obesity, both of which would be contribute to improved fetal nutrition. Similarly, a previous successful pregnancy would be associated with potential maternal weight retention and thereby increased fetal weight in subsequent pregnancies [34].

We examined the associates of preterm birth (<37 weeks the established definition of preterm). Although it is possible that obstetricians may consider IVF pregnancies as precious and have a lower threshold for iatrogenic preterm birth, we think this is unlikely because of the established associations of prematurity with neonatal respiratory complications [35],[36], and our finding that similar associations were also found for extreme preterm birth support this assumption. In the UK since 2005 only two embryos are allowed to be replaced under the age of 40 to reduce the risks of preterm birth and low birth weight, which are associated with multiple pregnancies. We have restricted the analysis of perinatal outcomes to delivery of a singleton pregnancy only because of the relevance of understanding risk factors associated with these outcomes in couples requiring IVF even when there is a singleton pregnancy. Few previous studies have examined the relationship of couple and treatment characteristics with gestational age and birth weight after live singleton IVF birth; our findings highlight important areas for further research aimed at maximising the success of IVF in terms of a healthy-weight, term live birth.

We demonstrate that a previous live birth as a consequence of IVF has an even greater effect on the prospect of successful assisted conception therapy than does previous spontaneous conception. Although many couples undergoing assisted conception feel encouraged by achieving a pregnancy, even if it subsequently results in fetal loss, we found no beneficial or negative effect of a history of a nonviable pregnancy on live birth. This suggests that embryonic chromosomal errors, rather than a defective maternal environment, may be primarily responsible.

Our work has a number of strengths. We have considered a range of anamnestic couple characteristics simultaneously with respect to validated live birth and perinatal outcomes rather than one or two in isolation. As a result, our data give a better overall reflection of predictive abilities, or lack thereof, for many factors. The size of our study was extremely large compared to other such studies in the literature. Finally, we considered a relevant multivariable historical model for consistency of findings before developing and assessing a novel prognostic model.

We acknowledge, however, a number of limitations. Data were not complete on 12% of the eligible cohort; however, univariable analysis was similar in the whole cohort, and multivariable multiple imputation did not alter the overall conclusions (results available from authors on request). Treatment cycles rather than individual patients were identified because of concerns regarding confidentiality and breach of the terms of the HFEA Act, and therefore it was not possible to examine the effect of multiple cycles within one patient or to use robust standard errors that take account of clustering of women. However, the previous HFEA analysis could account for clustering and did not show a significant effect as compared with per treatment cycle [7]. Maternal age was supplied in categories because of recent concerns over confidentiality; however, our findings of a decline in live birth rates with increasing age are in keeping with the previous analysis of the HFEA database and population reports [1],[7],[37]. We accept that the cause of infertility may have been underinvestigated or misreported [38], although for male factors this was cross-validated with the use of ICSI, and for tubal disease our observed decrease in success rate is consistent with the control arm of randomised controlled trials of salpingectomy prior to IVF [39], suggesting that these data are accurate.

For the main analyses with successful birth as the outcome we included both single and multiple pregnancy, i.e. our outcome was at least one live birth irrespective of whether there was one or more heart beats at 8 wk. Our reasons for doing this were, first, that this is a relevant outcome for infertile couples and, second, this was the outcome used in the study that developed the established prediction model, and therefore we wanted to test this model with the same outcome. Note that restriction of the data to pregnancies in which only one fetal sac was evident at 8 wk gestation produced similar results.

Finally, we acknowledge lack of external validation of our model. Nonetheless, we believe that this model will improve the ability to stratify contemporary couples seeking IVF on the basis of low, moderate, or high likelihood of success. This is of particular relevance to couples willing to consider all therapeutic options, including use of donor oocytes, as there is a 5-fold difference in live birth between the lowest and highest decile of our prediction model. To facilitate validation of the model we are currently generating a free web-based prediction tool (http://www.IVFpredict.com) and iPhone/Android application (IVFpredict) for widespread use of our new prediction tool. These will acknowledge the current lack of external validation and will request provision of anonymised data (all variables included in the prediction model, country of treatment, and outcome) that in the coming years we will use as a means of external validation of this model. We have included full model details in Text S2 thereby facilitating model validation by other research groups.

In conclusion, we show that baseline couple and treatment characteristics can provide a basis for counselling and informing couples of their likely prognosis in terms of low, moderate, or high odds of success (see Table 4).

## Supporting Information

Figure S1 Rates of successful live birth outcome per IVF treatment cycle by year of treatment. *N* = 163,425 (eligible cohort) cycles of IVF treatment in the United Kingdom.
(0.13 MB TIF)

Table S1 Description of cohort of IVF treatment rounds. *N* = 163,425 eligible participants.
(0.08 MB DOC)

Table S2 Univariable associations of potential predictors for live birth following IVF. *N* = 163,425 eligible; numbers for each predictor vary due to some missing data.
(0.09 MB DOC)

Table S3 Associations of duration of infertility and source of oocyte with live birth, stratified by maternal age.
(0.04 MB DOC)

Table S4 Associations of causes of infertility and number of treatment cycles with live birth, stratified by use of ICSI.
(0.03 MB DOC)

Table S5 Associations of potential predictors for live birth following IVF with multiple pregnancies removed.
(0.07 MB DOC)

Table S6 Univariable associations of potential risk factors with preterm birth, low birth weight, and macrosomia amongst singleton births following IVF.
(0.08 MB DOC)

Table S7 Univariable and multivariable associations of potential risk factors with extreme preterm birth (<33 wk) amongst singleton births following IVF.
(0.08 MB DOC)

Text S1 Calculation of predictive probability of live birth after IVF according to Templeton model.
(0.03 MB DOC)

Text S2 Equation and tables for derivation of probability of live birth.
(0.02 MB DOC)

## Abbreviations

AUROC: area under the curve of receiver operator characteristics
CI: confidence interval
HFEA: UK Human Fertilisation and Embryology Authority
ICSI: intracytoplasmic sperm injection
IDI: integrated discrimination improvement
IVF: in vitro fertilisation

## References

[1] Nyboe Andersen A, Goossens V, Bhattacharya S, Ferraretti AP, Kupka MS (2009). Assisted reproductive technology and intrauterine inseminations in Europe, 2005: results generated from European registers by ESHRE: ESHRE. The European IVF Monitoring Programme (EIM), for the European Society of Human Reproduction and Embryology (ESHRE).. Hum Reprod.

[2] Centers for Disease Control and Prevention ASfRM, Society for Assisted Reproductive Technology (2008). 2006 Assisted reproductive technology success rates: National summary and fertility clinic reports.. http://www.cdc.gov/art/art2006/index.htm.

[3] Australian Government Department of Health and Ageing (2006). Report of the independent review of assisted reproductive technologies.. http://www.health.gov.au/internet/main/publishing.nsf/Content/ART-Report.

[4] Leushuis E, van der Steeg JW, Steures P, Bossuyt PMM, Eijkemans MJC (2009). Prediction models in reproductive medicine: A critical appraisal.. Hum Reprod Update.

[5] Stolwijk AM, Zielhuis GA, Hamilton CJCM, Straatman H, Hollanders JMG (1996). Pregnancy: Prognostic models for the probability of achieving an ongoing pregnancy after in-vitro fertilization and the importance of testing their predictive value.. Hum Reprod.

[6] Stolwijk AM, Straatman H, Zielhuis GA, Jansen CA, Braat DD (1998). External validation of prognostic models for ongoing pregnancy after in- vitro fertilization.. Hum Reprod.

[7] Templeton A, Morris JK, Parslow W (1996). Factors that affect outcome of in-vitro fertilisation treatment.. Lancet.

[8] Hunault CC, Eijkemans MJ, Pieters MH, te Velde ER, Habbema JD (2002). A prediction model for selecting patients undergoing in vitro fertilization for elective single embryo transfer.. Fertil Steril.

[9] Hunault CC, te Velde ER, Weima SM, Macklon NS, Eijkemans MJ (2007). A case study of the applicability of a prediction model for the selection of patients undergoing in vitro fertilization for single embryo transfer in another center.. Fertil Steril.

[10] Evers JL (2002). Female subfertility.. Lancet.

[11] Vayena E, Rowe PJ, Griffin PD, WHO (2002). Current practices and controversies in assisted reproduction: Report of a WHO Meeting.. Medical, Ethical and Social Aspects of Assisted Reproduction.

[12] Min JK, Breheny SA, MacLachlan V, Healy DL (2004). What is the most relevant standard of success in assisted reproduction? The singleton, term gestation, live birth rate per cycle initiated: The BESST endpoint for assisted reproduction.. Hum Reprod.

[13] Barker DJ Fetal and Infant origins of Adult Disease..

[14] Jackson RA, Gibson KA, Wu YW, Croughan MS (2004). Perinatal outcomes in singletons following in vitro fertilization: A meta-analysis.. Obstet Gynecol.

[15] Allen VM, Wilson RD, Cheung A (2006). Pregnancy outcomes after assisted reproductive technology.. J Obstet Gynaecol Can.

[16] Draper ES, Kurinczuk JJ, Abrams KR, Clarke M (1999). Assessment of separate contributions to perinatal mortality of infertility history and treatment: A case-control analysis.. Lancet.

[17] Romundstad LB, Romundstad PR, Sunde A, von During V, Skjaerven R (2008). Effects of technology or maternal factors on perinatal outcome after assisted fertilisation: A population-based cohort study.. Lancet.

[18] Basso O, Baird DD (2003). Infertility and preterm delivery, birthweight, and caesarean section: A study within the Danish National Birth Cohort.. Hum Reprod.

[19] Nygren KG, Finnstrom O, Kallen B, Olausson PO (2007). Population-based Swedish studies of outcomes after in vitro fertilisation.. Acta Obstet Gynecol Scand.

[20] von Elm E, Altman DG, Egger M, Pocock SJ, Gøtzsche PC (2007). The Strengthening the Reporting of Observational Studies in Epidemiology (STROBE) statement: Guidelines for reporting observational studies.. Lancet.

[21] DeLong ER, DeLong DM, Clarke-Pearson DL (1988). Comparing the areas under two or more correlated receiver operating characteristic curves: A nonparametric approach.. Biometrics.

[22] Pencina MJ, D'Agostino RB, D'Agostino RB, Vasan RS (2008). Evaluating the added predictive ability of a new marker: From area under the ROC curve to reclassification and beyond.. Stat Med.

[23] Smeenk JMJ, Stolwijk AM, Kremer JAM, Braat DDM (2000). External validation of the Templeton model for predicting success after IVF.. Hum Reprod.

[24] Palermo G, Joris H, Devroey P, Van Steirteghem AC (1992). Pregnancies after intracytoplasmic injection of single spermatozoon into an oocyte.. Lancet.

[25] Mook-Kanamori DO, Steegers EA, Eilers PH, Raat H, Hofman A (2010). Risk factors and outcomes associated with first-trimester fetal growth restriction.. JAMA.

[26] Smith GC, Smith MF, McNay MB, Fleming JE (1998). First-trimester growth and the risk of low birth weight.. N Engl J Med.

[27] Bukowski R, Smith GCS, Malone FD, Ball RH, Nyberg DA (2007). Fetal growth in early pregnancy and risk of delivering low birth weight infant: Prospective cohort study.. BMJ.

[28] Colditz GA, Willett WC, Stampfer MJ, Rosner B, Speizer FE (1987). Menopause and the risk of coronary heart disease in women.. N Engl J Med.

[29] van der Schouw YT, van der Graaf Y, Steyerberg EW, Eijkemans JC, Banga JD (1996). Age at menopause as a risk factor for cardiovascular mortality.. Lancet.

[30] Cooley M, Bakalov V, Bondy CA (2003). Lipid profiles in women with 45,X vs 46,XX primary ovarian failure.. JAMA.

[31] Redman CW, Sargent IL (2003). Pre-eclampsia, the placenta and the maternal systemic inflammatory response–A review.. Placenta.

[32] Bodri D, Vernaeve V, Figueras F, Vidal R, Guillen JJ (2006). Oocyte donation in patients with Turner's syndrome: A successful technique but with an accompanying high risk of hypertensive disorders during pregnancy.. Hum Reprod.

[33] Paulson RJ, Boostanfar R, Saadat P, Mor E, Tourgeman DE (2002). Pregnancy in the sixth decade of life: Obstetric outcomes in women of advanced reproductive age.. JAMA.

[34] Villamor E, Cnattingius Cnattingius Interpregnancy weight change and risk of adverse pregnancy outcomes: A population-based study.. Lancet.

[35] Morrison JJ, Rennie JM, Milton PJ (1995). Neonatal respiratory morbidity and mode of delivery at term: Influence of timing of elective caesarean section.. Br J Obstet Gynaecol.

[36] Stutchfield P, Whitaker R, Russell I (2005). Antenatal betamethasone and incidence of neonatal respiratory distress after elective caesarean section: Pragmatic randomised trial.. BMJ.

[37] Nyboe Andersen A, Goossens V, Ferraretti AP, Bhattacharya S, Felberbaum R (2008). Assisted reproductive technology in Europe, 2004: Results generated from European registers by ESHRE.. Hum Reprod.

[38] Craft I, Forman R (1997). Analysis of IVF data.. Lancet.

[39] Johnson N, van Voorst S, Sowter MC, Strandell A, Mol BW (2010). Surgical treatment for tubal disease in women due to undergo in vitro fertilisation.. Cochrane Database Syst Rev.

