# Supplementary figures and images for: Predicting Live Birth, Preterm Delivery, and Low Birth Weight in Infants Born from In Vitro Fertilisation: A Prospective Study of 144,018 Treatment Cycles
Source: PLoS Med. 2011 Jan 4;8(1):e1000386. doi: 10.1371/journal.pmed.1000386 (PMC3014925; doi:10.1371/journal.pmed.1000386)

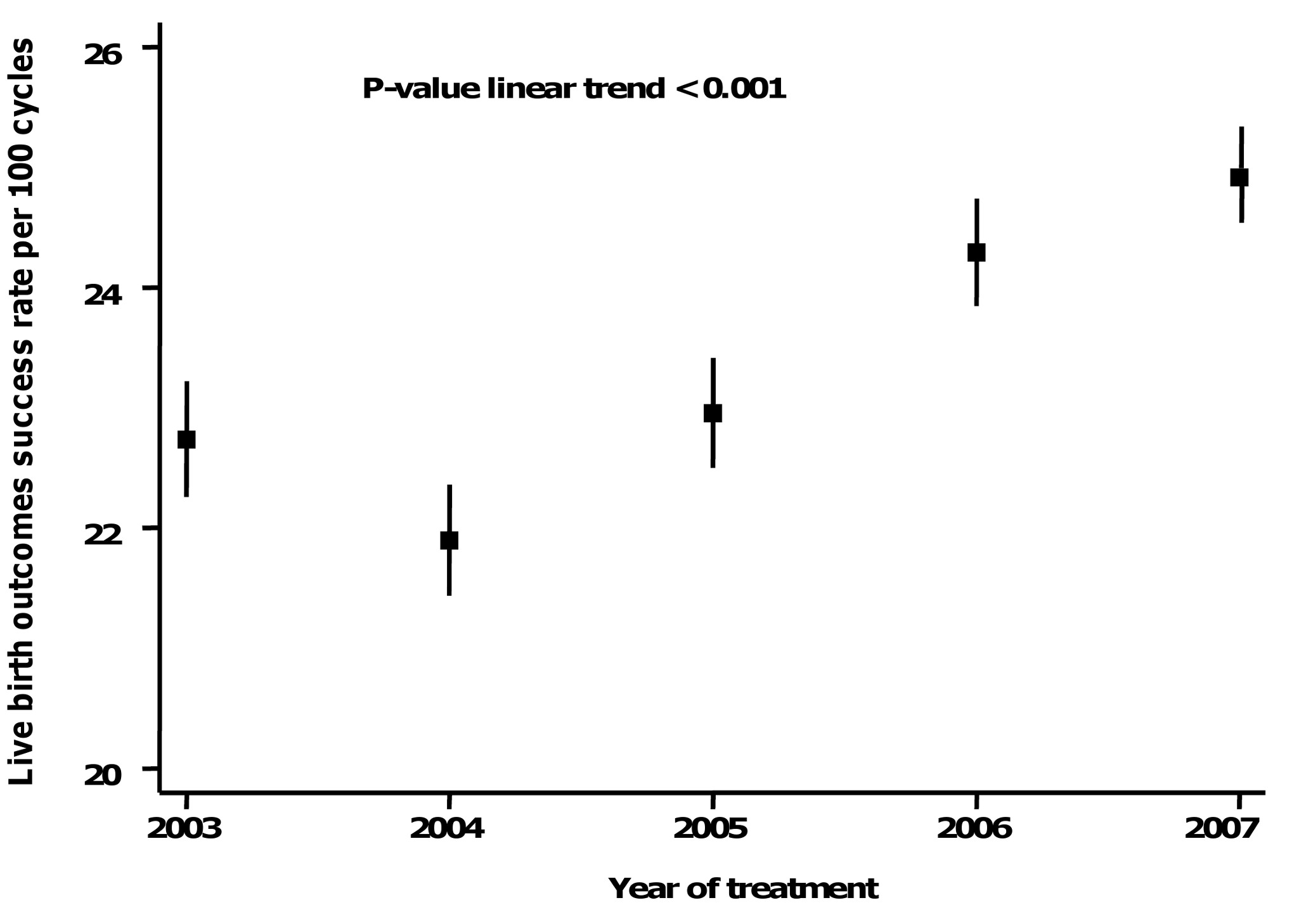

Supplement: Figure S1 — Rates of successful live birth outcome per IVF treatment cycle by year of treatment. N = 163,425 (eligible cohort) cycles of IVF treatment in the United Kingdom. (0.13 MB TIF) [file pmed.1000386.s001.tif]
