# Supplementary material for: Predicting Live Birth, Preterm Delivery, and Low Birth Weight in Infants Born from In Vitro Fertilisation: A Prospective Study of 144,018 Treatment Cycles
Source: PLoS Med. 2011 Jan 4;8(1):e1000386. doi: 10.1371/journal.pmed.1000386 (PMC3014925; doi:10.1371/journal.pmed.1000386)
Supplement: Table S1 — Description of cohort of IVF treatment rounds. N = 163,425 eligible participants. (0.08 MB DOC) [file pmed.1000386.s002.doc]

**Table S1: Description of cohort of IVF treatment rounds. N = 163,425 eligible participants**

| **Characteristic** | **Categories** | **N (%) with missing** | **N (%) for categories** | **Mean (SD) for continuous variables** |
| --- | --- | --- | --- | --- |
| Year of treatment | 2003 | 0 | 28788 (17.6) |  |
|  | 2004 |  | 30721 (18.8) |  |
|  | 2005 |  | 32166 (19.7) |  |
|  | 2006 |  | 34938 (21.4) |  |
|  | 2007 |  | 36812 (22.5) |  |
| **Potential predictors** | | | | |
| Maternal age (years) | 18-34 | 218 (0.2) | 68008 (41.7) |  |
|  | 35-37 |  | 40984 (25.1) |  |
|  | 38-39 |  | 24837 (15.2) |  |
|  | 40-42 |  | 21218 (13.0) |  |
|  | 43-44 |  | 5334 (3.3) |  |
|  | 45-50 |  | 2763 (1.7) |  |
|  | Continuous years |  |  | 33.3 (6.5) |
| Duration of infertility (years) | <1 | 13712 (8.4) | 1799 (1.2) |  |
|  | 1-3 |  | 50278 (33.6) |  |
|  | 4-6 |  | 54738 (36.6) |  |
|  | 7-9 |  | 22173 (14.8) |  |
|  | 9-12 |  | 9506 (6.4) |  |
|  | >12 |  | 11219 (7.5) |  |
|  | Continuous years |  |  | 5.6 (4.1) |
| Cause of infertility | Unknown | 0 | 44409 (27.2) |  |
|  | Tubal only |  | 24734 (15.1) |  |
|  | Anovulatory only |  | 15304 (9.4) |  |
|  | Endometriosis only |  | 5463 (3.3) |  |
|  | Cervical only |  | 76 (0.1) |  |
|  | Male only |  | 57060 (34.9) |  |
|  | Combination known causes |  | 16379 (10.0) |  |
| Number of previous unsuccessful IVF | 0 | 0 | 146800 (89.8) |  |
|  | 1 |  | 8518 (5.2) |  |
|  | 2 |  | 3984 (2.4) |  |
|  | 3 |  | 1956 (1.2) |  |
|  | 4 |  | 1073 (0.7) |  |
|  | >=5 |  | 1094 (0.7) |  |

Table S1: Continued

| **Characteristic** | **Categories** | **N (%) with missing** | **N (%) for categories** | **Mean (SD) for continuous variables** |
| --- | --- | --- | --- | --- |
| Mutually exclusive categories of previous IVF and obstetric history | No previous IVF, no previous pregnancy | 0 | 94348 (57.7) |  |
|  | No previous IVF, at least one previous pregnancy, no live births |  | 30556 (18.7) |  |
|  | No previous IVF, at least one previous pregnancy, at least one live birth |  | 15434 (9.4) |  |
|  | Previous IVF, no previous pregnancy |  | 10619 (6.5) |  |
|  | Previous IVF, at least one previous pregnancy, no live birth |  | 6965 (4.2) |  |
|  | Previous IVF, at least one previous pregnancy, at least one live birth |  | 5503 (3.4) |  |
| Type of hormonal preparation | Antioestrogens | 6296 (3.9) | 1708 (1.1) |  |
|  | Gonadatropins |  | 150328 (95.7) |  |
|  | Hormone Replacement |  | 5093 (3.2) |  |
| Number of treatment cycles | 1 | 0 | 93795 (57.4) |  |
|  | 2 |  | 34860 (21.3) |  |
|  | >=3 |  | 34770 (21.3) |  |
| Source of egg | Donor | 0 | 6639 (4.1) |  |
|  | Patient |  | 156786 (95.9) |  |
| Treatment type | IVF | 0 | 88244 (54.0) |  |
|  | IVF plus ICSI |  | 75181 (46.0) |  |
| **Outcomes** | | | | |
| Number of live births | 0 | 0 | 125109 (76.6) |  |
|  | 1 |  | 29054 (17.8) |  |
|  | 2 |  | 9134 (5.6) |  |
|  | 3 |  | 128 (0.1) |  |

IVF: in vitro fertilisation; ICSI: Intracytoplasmic sperm injection; N: numbe
