# Supplementary material for: Predicting Live Birth, Preterm Delivery, and Low Birth Weight in Infants Born from In Vitro Fertilisation: A Prospective Study of 144,018 Treatment Cycles
Source: PLoS Med. 2011 Jan 4;8(1):e1000386. doi: 10.1371/journal.pmed.1000386 (PMC3014925; doi:10.1371/journal.pmed.1000386)
Supplement: Table S2 — Univariable associations of potential predictors for live birth following IVF. N = 163,425 eligible; numbers for each predictor vary due to some missing data. (0.09 MB DOC) [file pmed.1000386.s003.doc]

**Table S2: Univariable associations of potential predictors for live birth following IVF. N = 163,425 eligible – numbers for each predictor vary due to some missing data**

| **Characteristic** | **Categories** | **Number** | **Rate of at least 1 live birth per 100 IVF treatments (95%CI)** | **Odds ratio of live birth (95%CI)** | **p-value*** |
| --- | --- | --- | --- | --- | --- |
| Maternal age (years) | 18-34 | 68008 | 29.7 (29.3, 30.0) | 1 | < 0.001 |
|  | 35-37 | 40984 | 24.5 (24.1, 24.9) | 0.77 (0.75, 0.79) |  |
|  | 38-39 | 24837 | 18.3 (17.9, 18.9) | 0.53 (0.51, 0.55) |  |
|  | 40-42 | 21218 | 11.9 (11.4, 12.3) | 0.32 (0.31, 0.33) |  |
|  | 43-44 | 5334 | 8.7 (8.0, 9.5) | 0.23 (0.21, 0.25) |  |
|  | 45-50 | 2763 | 16.9 (15.6, 18.3) | 0.48 (0.44, 0.53) |  |
| Duration of infertility (years) | <1 | 1799 | 31.9 (29.8, 34.0) | 1.51 (1.37, 1.67) | < 0.001 |
|  | 1-3 | 50278 | 25.3 (24.9, 25.7) | 1.10 (1.06, 1.13) |  |
|  | 4-6 | 54738 | 23.6 (23.3, 24.0) | 1 |  |
|  | 7-9 | 22173 | 22.0 (21.5, 22.6) | 0.91 (0.88, 0.95) |  |
|  | 9-12 | 9506 | 20.0 (19.2, 20.8) | 0.81 (0.76, 0.85) |  |
|  | >12 | 11219 | 18.3 (17.6, 19.1) | 0.72 (0.69, 0.76) |  |
| Cause of infertility | Unknown | 44409 | 22.7 (22.2, 23.1) | 1 | < 0.001 |
|  | Tubal only | 24734 | 21.4 (20.9, 21.9) | 0.93 (0.90, 0.97) |  |
|  | Anovulatory only | 15304 | 22.8 (22.1, 23.5) | 1.01 (0.96, 1.05) |  |
|  | Endometriosis only | 5463 | 23.6 (22.5, 24.7) | 1.05 (0.98, 1.12) |  |
|  | Cervical only | 76 | 10.5 (5.3, 19.7) | 0.40 (0.19, 0.84) |  |
|  | Male only | 57060 | 25.3 (24.9, 25.7) | 1.15 (1.12, 1.19) |  |
|  | Combination known causes | 16379 | 22.8 (22.1, 23.4) | 1.01 (0.96, 1.05) |  |
| Number of previous unsuccessful IVF | 0 | 146800 | 24.0 (23.8, 22.4) | 1 | < 0.001 |
|  | 1 | 8518 | 19.3 (18.5, 20.2) | 0.76 (0.72, 0.80) |  |
|  | 2 | 3984 | 18.2 (17.1, 19.5) | 0.71 (0.65, 0.77) |  |
|  | 3 | 1956 | 20.2 (18.5, 22.0) | 0.80 (0.72, 0.90) |  |
|  | 4 | 1073 | 13.9 (11.9, 16.1) | 0.51 (0.43, 0.61) |  |
|  | >=5 | 1094 | 17.7 (15.6, 20.1) | 0.68 (0.58, 0.80) |  |

**Table S2: Continued**

| **Characteristic** | **Categories** | **Number** | **Rate of at least 1 live birth per 100 IVF treatments (95%CI)** | **Odds ratio of live birth (95%CI)** | **p-value*** |
| --- | --- | --- | --- | --- | --- |
| Mutually exclusive categories of previous IVF and obstetric history | No previous IVF, no previous pregnancy | 94348 | 22.4 (24.1, 24.7) | 1 | < 0.001 |
|  | No previous IVF, at least 1 pregnancy, 0 live births | 30556 | 22.4 (21.9, 22.9) | 0.89 (0.87, 0.92) |  |
|  | No previous IVF, at least 1 pregnancy, at least 1 live birth | 15434 | 23.2 (22.5, 23.9) | 0.94 (0.89, 0.97) |  |
|  | Previous IVF, 0 pregnancy | 10619 | 19.4 (18.6, 20.1) | 0.74 (0.71, 0.78) |  |
|  | Previous IVF, at least 1 pregnancy, 0 live birth | 6965 | 18.6 (17.7, 19.6) | 0.79 (0.73, 0.87) |  |
|  | Previous IVF, at least 1 pregnancy, at least 1 live birth | 5503 | 27.3 (26.2, 28.5) | 0.71 (0.67, 0.75) |  |
| Type of hormonal preparation | Antioestrogens | 1708 | 19.3 (17.5, 21.3) | 1 | < 0.001 |
|  | Gonadatropins | 150328 | 23.3 (23.1, 23.5) | 1.26 (1.12, 1.43) |  |
|  | Hormone Replacement | 5093 | 26.1 (24.9, 27.4) | 1.48 (1.29, 1.69) |  |
| Cycle number | 1 | 93795 | 25.1 (24.9, 25.4) | 1 | < 0.001 |
|  | 2 | 34860 | 21.4 (21.0, 21.9) | 0.81 (0.79, 0.84) |  |
|  | >=3 | 34770 | 20.9 (20.5, 21.3) | 0.79 (0.76, 0.81) |  |
| Source of egg | Donor | 6639 | 28.1 (27.0, 29.2) | 1 | < 0.001 |
|  | Patient | 156786 | 23.2 (23.0, 23.5) | 0.78 (0.73, 0.82) |  |
| Type of treatment | IVF | 88244 | 21.6 (21.3, 21.9) | 1 | < 0.001 |
|  | IVF plus ICSI | 75181 | 25.6 (25.3, 25.9) | 1.25 (1.22, 1.28) |  |

* All p-values are likelihood ratio tests of null hypothesis that the odds are the same for each category (i.e. they do not assume linearity)
