# Supplementary material for: Predicting Live Birth, Preterm Delivery, and Low Birth Weight in Infants Born from In Vitro Fertilisation: A Prospective Study of 144,018 Treatment Cycles
Source: PLoS Med. 2011 Jan 4;8(1):e1000386. doi: 10.1371/journal.pmed.1000386 (PMC3014925; doi:10.1371/journal.pmed.1000386)
Supplement: Table S3 — Associations of duration of infertility and source of oocyte with live birth, stratified by maternal age. (0.04 MB DOC) [file pmed.1000386.s004.doc]

**Table S3: Associations of duration of infertility and source of oocyte with live birth, stratified by maternal age**

| **Characteristic** | **Categories of characteristics** | **Odds ratio (95%CI) of association with live birth by categories of maternal age (Years):** | | | | |
| --- | --- | --- | --- | --- | --- | --- |
|  |  | **18-34** | **35-37** | **38-39** | **40-42** | **>=43** |
| **Duration of infertility (years)** | <1 | 1.50 (1.29, 1.74) | 1.49 (1.92, 1.86) | 1.64 (1.23, 2.19) | 1.70 (1.21, 2.40) | -* |
|  | 1-3 | 1.14 (1.10, 1.19) | 1.10 (1.03, 1.16) | 1.05 (0.96, 1.14) | 1.07 (0.95, 1.20) | 1.02 (0.73, 1.42) |
|  | 4-6 | 1 | 1 | 1 | 1 | 1 |
|  | 7-9 | 0.92 (0.87, 0.97) | 0.97 (0.90, 1.05) | 0.96 (0.86, 1.07) | 0.96 (0.82, 1.12) | 1.10 (0.74, 1.65) |
|  | 9-12 | 0.86 (0.79, 0.93) | 0.86 (0.79, 0.96) | 0.91 (0.78, 1.05) | 0.92 (0.75, 1.13) | 0.55 (0.29, 1.07) |
|  | >12 | 0.86 (0.78, 0.96) | 0.89 (0.78, 0.96) | 0.97 (0.86, 1.11) | 0.86 (0.72, 1.03) | 0.60 (0.37, 0.96) |
| **Source of egg** | Donor | 1 | 1 | 1 | 1 | 1 |
|  | Patient | 1.04 (0.69, 1.54) | 0.63 (0.40, 0.99) | 0.71 (0.43, 1.14) | 0.29 (0.20, 0.42) | 0.11 (0.79, 1.44) |

* No observations in this category
