# Supplementary material for: Predicting Live Birth, Preterm Delivery, and Low Birth Weight in Infants Born from In Vitro Fertilisation: A Prospective Study of 144,018 Treatment Cycles
Source: PLoS Med. 2011 Jan 4;8(1):e1000386. doi: 10.1371/journal.pmed.1000386 (PMC3014925; doi:10.1371/journal.pmed.1000386)
Supplement: Table S4 — Associations of causes of infertility and number of treatment cycles with live birth, stratified by use of ICSI. (0.03 MB DOC) [file pmed.1000386.s005.doc]

**Table S4: Associations of causes of infertility and number of treatment cycles with live birth, stratified by use of ICSI.**

| **Characteristic** | **Categories of characteristic** | **Odds ratio (95%CI) of association with live birth by categories of whether ICSI used or not:** | |
| --- | --- | --- | --- |
|  |  | **ICSI** | **No ICSI** |
| **Main cause of infertility** | Unknown | 1 | 1 |
|  | Tubal | 0.80 (0.72, 0.88) | 0.86 (0.83, 0.91) |
|  | Anovulatory | 1.02 (0.92, 1.13) | 0.92 (0.87, 0.98) |
|  | Endometriosis | 0.95 (0.81, 1.11) | 0.96 (0.88, 1.04) |
|  | Cervical | 1.30 (0.23, 7.40) | 0.32 (0.14, 0.73) |
|  | Male | 1.02 (0.97, 1.08) | 0.76 (0.72, 0.81) |
|  | Combination | 0.99 (0.93, 1.07) | 0.80 (0.61, 0.83) |
| **Number of treatment cycles** | 1 | 1 | 1 |
|  | 2 | 0.84 (0.81, 0.88) | 0.85 (0.81, 0.89) |
|  | >=3 | 0.82 (0.78, 0.86) | 0.97 (0.92, 1.01) |
