# Supplementary material for: Predicting Live Birth, Preterm Delivery, and Low Birth Weight in Infants Born from In Vitro Fertilisation: A Prospective Study of 144,018 Treatment Cycles
Source: PLoS Med. 2011 Jan 4;8(1):e1000386. doi: 10.1371/journal.pmed.1000386 (PMC3014925; doi:10.1371/journal.pmed.1000386)
Supplement: Table S5 — Associations of potential predictors for live birth following IVF with multiple pregnancies removed. (0.07 MB DOC) [file pmed.1000386.s006.doc]

**Table S5: Associations of potential predictors for live birth following IVF with multiple pregnancies removed.
N = 134,087 analysis cohort with complete data on all variables included in any model and after removal of cycles in which two or more heart beats were detected at 8 weeks gestation**

| **Characteristic** | **Categories** | **Univariable odds ratio of live birth (95%CI)** | **Multivariable^a^ odds ratio of live birth (95%CI)** | **p-value^b^** |
| --- | --- | --- | --- | --- |
| Maternal age (years) | 18-34 | 1 | 1 | < 0.001 |
|  | 35-37 | 0.83 (0.80, 0.86) | 0.85 (0.82, 0.88) |  |
|  | 38-39 | 0.61 (0.58, 0.63) | 0.62 (0.59, 0.65) |  |
|  | 40-42 | 0.34 (0.33, 0.36) | 0.35 (0.33, 0.37) |  |
|  | 43-44 | 0.14 (0.12, 0.16) | 0.14 (0.12, 0.16) |  |
|  | 45-50 | 0.15 (0.12, 0.20) | 0.13 (0.10, 0.18) |  |
| Duration of infertility (years) | <1 | 1.52 (1.36, 1.71) | 1.55 (1.37, 1.74) | < 0.001 |
|  | 1-3 | 1.09 (1.06, 1.13) | 1.10 (1.07, 1.15) |  |
|  | 4-6 | 1 | 1 |  |
|  | 7-9 | 0.93 (0.89, 0.97) | 0.96 (0.91, 0.99) |  |
|  | 9-12 | 0.82 (0.77, 0.87) | 0.88 (0.82, 0.93) |  |
|  | >12 | 0.72 (0.68, 0.77) | 0.89 (0.83, 0.95) |  |
| Cause of infertility | Unknown | 1 | 1 | < 0.001 |
|  | Tubal only | 0.94 (0.90, 0.98) | 0.89 (0.85, 0.93) |  |
|  | Anovulatory only | 0.92 (0.87, 0.97) | 0.95 (0.90, 1.01) |  |
|  | Endometriosis only | 1.04 (0.96, 1.13) | 0.96 (0.89, 1.04) |  |
|  | Cervical only | 0.49 (0.23, 1.07) | 0.47 (0.22, 1.04) |  |
|  | Male only | 1.16 (1.12, 1.20) | 0.91 (0.88, 0.95) |  |
|  | Combination known causes | 0.99 (0.94, 1.05) | 0.87 (0.83, 0.92) |  |
| Number of previous unsuccessful IVF | 0 | 1 | 1 | < 0.001 |
|  | 1 | 0.74 (0.69, 0.80) | 0.70 (0.62, 0.80) |  |
|  | 2 | 0.68 (0.62, 0.75) | 0.67 (0.58, 0.77) |  |
|  | 3 | 0.72 (0.62, 0.83) | 0.72 (0.60, 0.86) |  |
|  | 4 | 0.51 (0.41, 0.64) | 0.52 (0.41, 0.67) |  |
|  | >=5 | 0.57 (0.46, 0.71) | 0.65 (0.51, 0.82) |  |
| Mutually exclusive categories of previous IVF and obstetric history | No previous IVF, 0 pregnancy | 1 | 1 | < 0.001 |
|  | No previous IVF, at least 1 pregnancy, 0 live births | 0.88 (0.85, 0.91) | 1.00 (0.96, 1.04) |  |
|  | No previous IVF, at least 1 pregnancy, at least 1 live birth | 0.93 (0.88, 0.97) | 1.16 (1.11, 1.22) |  |
|  | Previous IVF, 0 pregnancy | 0.72 (0.67, 0.76) | 1.16 (1.01, 1.32) |  |
|  | Previous IVF, at least 1 pregnancy, 0 live birth | 0.68 (0.63, 0.74) | 1.01 (0.91, 1.12) |  |
|  | Previous IVF, at least 1 pregnancy, at least 1 live birth | 1.12 (1.04, 1.21) | 1.58 (1.45, 1.71) |  |

| **Characteristic** | **Categories** | **Univariable odds ratio of live birth (95%CI)** | **Multivariable^a^ odds ratio of live birth (95%CI)** | **p-value^b^** |
| --- | --- | --- | --- | --- |
| Hormonal preparation | Antioestrogen | 1 | 1 | < 0.001 |
|  | Gonadatropin | 1.36 (1.17, 1.59) | 1.24 (1.06, 1.46) |  |
|  | Hormone replacement | 1.55 (1.30, 1.85) | 1.44 (1.20, 1.73) |  |
| Cycle number | 1 | 1 | 1 | < 0.001 |
|  | 2 | 0.84 (0.81, 0.87) | 0.87 (0.84, 0.91) |  |
|  | >=3 | 0.79 (0.76, 0.81) | 0.88 (0.85, 0.92) |  |
| Source of egg | Donor | 1 | 1 | < 0.001 |
|  | Patient | 0.94 (0.78, 1.14) | 0.46 (0.37, 0.56) |  |
| Treatment type | IVF | 1 | 1 | < 0.001 |
|  | ICSI plus IVF | 1.30 (1.27, 1.34) | 1.31 (1.26, 1.35) |  |

^a^ Multivariable adjusted = mutual adjustment for all variables listed in column one

^b^ P-value for multivariable association; all p-values are likelihood ratio tests of null hypothesis that the odds are the same for each category (i.e. they do not assume linearity)
