# Supplementary material for: Predicting Live Birth, Preterm Delivery, and Low Birth Weight in Infants Born from In Vitro Fertilisation: A Prospective Study of 144,018 Treatment Cycles
Source: PLoS Med. 2011 Jan 4;8(1):e1000386. doi: 10.1371/journal.pmed.1000386 (PMC3014925; doi:10.1371/journal.pmed.1000386)
Supplement: Table S6 — Univariable associations of potential risk factors with preterm birth, low birth weight, and macrosomia amongst singleton births following IVF. (0.08 MB DOC) [file pmed.1000386.s007.doc]

**Table S6: Univariable associations of potential risk factors with preterm birth, low birth weight and macrosomia amongst singleton births following IVF.**

| **Characteristic** | **Categories** | **Univariable association with preterm birth. N = 24096 in analyses with n = 2070 cases of preterm birth** | | **Univariable association with low birth weight. N = 21804 in analyses with n = 2100 cases of low birth weight** | | **Univariable association with low birth weight. N = 21950 in analyses with n = 2246 case of macrosomia** | |
| --- | --- | --- | --- | --- | --- | --- | --- |
|  |  | **Odds ratio (95%CI)** | **p-value** | **Odds ratio (95%CI)** | **p-value** | **Odds ratio (95%CI)** | **p-value** |
| Age (years) | 18-34 | 1 | 0.25 | 1 | 0.04 | 1 | < 0.001 |
|  | 35-37 | 0.92 (0.82, 1.01) |  | 0.88 (0.79, 0.98) |  | 1.18 (1.06, 1.30) |  |
|  | 38-39 | 0.90 (0.78, 1.04) |  | 0.86 (0.75, 0.99) |  | 1.13 (1.00, 1.30) |  |
|  | >=40 | 1.01 (0.86, 1.21) |  | 1.01 (0.85, 1.19) |  | 1.33 (1.13, 1.56) |  |
| Duration of infertility (years) | <1 | 1.14 (0.81, 1.62) | 0.002 | 0.90 (0.62, 1.30) | 0.04 | 0.87 (0.61, 1.24) | 0.05 |
|  | 1-3 | 0.99 (0.88, 1.10) |  | 0.93 (0.84, 1.03) |  | 0.90 (0.81, 0.99) |  |
|  | 4-6 | 1 |  | 1 |  |  |  |
|  | 7-9 | 1.17 (1.01, 1.34) |  | 1.06 (0.92, 1.22) |  | 1.06 (0.93, 1.21) |  |
|  | >=9 | 1.29 (1.12, 1.49) |  | 1.16 (1.00, 1.34) |  | 1.05 (0.90, 1.21) |  |
| Cause | Unknown | 1 | < 0.001 | 1 | 0.001 | 1 | 0.009 |
|  | Tubal only | 1.27 (1.11, 1.47) |  | 1.15 (1.00, 1.33) |  | 1.08 (0.94, 1.25) |  |
|  | Anovulatory only | 1.21 (1.01, 1.44) |  | 1.26 (1.06, 1.51) |  | 1.03 (0.86, 1.24) |  |
|  | Endometriosis only | 0.86 (0.65, 1.14) |  | 1.05 (0.82, 1.37) |  | 0.92 (0.71, 1.20) |  |
|  | Cervical only | 8.18 (1.83, 36.65) |  | 14.67 (2.45, 87.98) |  | 8.28 (1.30, 66.01) |  |
|  | Male only | 0.89 (0.79, 1.00) |  | 0.96 (0.85, 1.08) |  | 1.14 (1.02, 1.28) |  |
|  | Combination known causes | 1.17 (1.00, 1.38) |  | 1.13 (0.96, 1.34) |  | 0.89 (0.75, 1.06) |  |
| Previous unsuccessful IVF (number | 0 | 1 | 0.90 | 1 | 0.25 | 1 | 0.64 |
|  | 1 | 1.05 (0.84, 1.30) |  | 1.21 (0.98, 1.49) |  | 0.87 (0.69, 1.09) |  |
|  | 2 | 1.10 (0.80, 1.53) |  | 1.17 (0.85, 1.61) |  | 0.93 (0.66, 1.30) |  |
|  | >=3 | 0.94 (0.65, 1.36) |  | 0.93 (0.65, 1.34) |  | 1.02 (0.72, 1.42) |  |

**Table S6: continued**

| Mutually exclusive categories of previous IVF and obstetric history | No previous IVF, 0 pregnancy | 1 | 0.006 | 1 | < 0.001 | 1 | 0.001 |
| --- | --- | --- | --- | --- | --- | --- | --- |
|  | No previous IVF, at least 1 pregnancy, 0 live births | 1.25 (1.12, 1.41) |  | 1.18 (1.04, 1.32) |  | 1.20 (1.07, 1.34) |  |
|  | No previous IVF, at least 1 pregnancy, at least 1 live birth | 1.07 (0.92, 1.25) |  | 0.86 (0.73, 1.03) |  | 1.28 (1.11, 1.48) |  |
|  | Previous IVF, 0 pregnancy | 1.14 (0.94, 1.40) |  | 1.23 (1.01, 1.49) |  | 0.95 (0.77, 1.17) |  |
|  | Previous IVF, at least 1 pregnancy, 0 live birth | 1.18 (0.93, 1.52) |  | 1.22 (0.96, 1.55) |  | 0.97 (0.75, 1.26) |  |
|  | Previous IVF, at least 1 pregnancy, at least 1 live birth | 0.98 (0.76, 1.25) |  | 0.82 (0.63, 1.07) |  | 1.31 (1.06, 1.62) |  |
| Hormonal preparation | Antioestrogen | 1 | 0.11 | 1 | 0.94 | 1 | 0.88 |
|  | Gonadatropin | 0.68 (0.44, 1.05) |  | 0.92 (0.56, 1.50) |  | 1.04 (0.63, 1.72) |  |
|  | Hormone replacement | 0.81 (0.49, 1.35) |  | 0.93 (0.53, 1.63) |  | 1.11 (0.63, 1.96) |  |
| Cycle number | 1 | 1 | 0.64 | 1 | 0.38 | 1 | 0.002 |
|  | 2 | 1.00 (0.90, 1.13) |  | 0.97 (0.87, 1.09) |  | 1.09 (0.98, 1.22) |  |
|  | >=3 | 0.95 (0.84, 1.07) |  | 0.92 (0.81, 1.04) |  | 1.22 (1.09, 1.36) |  |
| Source of egg | Donor | 1 | < 0.001 | 1 | < 0.001 | 1 | 0.09 |
|  | Patient | 0.38 (0.25, 0.57) |  | 0.40 (0.26, 0.63) |  | 0.64 (0.38, 1.07) |  |
| Treatment type | IVF | 1 | 0.02 | 1 | 0.004 | 1 | 0.58 |
|  | IVF & ICSI | 0.83 (0.76, 0.91) |  | 0.88 (0.80, 0.96) |  | 1.02 (0.94, 1.12) |  |

Cycles include in analyses are those with complete data on all variables and who experienced a singleton birth after IVF. For associations with low birth weight, those with macrosomia as outcome are removed so that low birth weight is compared with normal birth weight and similarly for macrosomia those with low birth weight are removed so that macrosomia is compared with normal birth weight. P-values are likelihood ratio tests of null hypothesis that the odds are the same for each category (i.e. they do not assume linearity)
