# Supplementary material for: Predicting Live Birth, Preterm Delivery, and Low Birth Weight in Infants Born from In Vitro Fertilisation: A Prospective Study of 144,018 Treatment Cycles
Source: PLoS Med. 2011 Jan 4;8(1):e1000386. doi: 10.1371/journal.pmed.1000386 (PMC3014925; doi:10.1371/journal.pmed.1000386)
Supplement: Table S7 — Univariable and multivariable associations of potential risk factors with extreme preterm birth (<33 wk) amongst singleton births following IVF. (0.08 MB DOC) [file pmed.1000386.s008.doc]

**Table S7: Univariable and multivariable associations of potential risk factors with extreme preterm birth (< 33 weeks) amongst singleton births following IVF.**

| **Characteristic** | **Categories** | **Univariable association with extreme preterm birth. N = 22498 in analyses with n = 472 cases of extreme preterm birth** | | **Multivariable association with extreme preterm birth. N = 22498 in analyses with n = 472 cases of extreme preterm birth** | |
| --- | --- | --- | --- | --- | --- |
|  |  | **Odds ratio (95%CI)** | **p-value** | **Odds ratio (95%CI)** | **p-value** |
| Age (years) | 18-34 | 1 | 0.05 | 1 | 0.06 |
|  | 35-37 | 1.10 (0.89, 1.36) |  | 1.05 (0.85, 1.03) |  |
|  | 38-39 | 0.73 (0.53, 1.01) |  | 0.67 (0.49, 0.94) |  |
|  | >=40 | 1.25 (0.90, 1.73) |  | 1.10 (0.78, 1.54) |  |
| Duration of infertility (years) | <1 | 1.02 (0.47, 2.19) | 0.01 | 0.93 (0.42, 2.04) | 0.02 |
|  | 1-3 | 1.07 (0.85, 1.33) |  | 1.05 (0.83, 1.31) |  |
|  | 4-6 | 1 |  | 1 |  |
|  | 7-9 | 1.24 (0.93, 1.64) |  | 1.24 (0.93, 1.65) |  |
|  | >=9 | 1.65 (1.25, 2.19) |  | 1.63 (1.21, 2.18) |  |
| Cause | Unknown | 1 | 0.002 | 1 | 0.02 |
|  | Tubal only | 1.18 (0.88, 1.56) |  | 1.10 (0.82, 1.47) |  |
|  | Anovulatory only | 1.52 (1.10, 2.11) |  | 1.46 (1.04, 2.03) |  |
|  | Endometriosis only | 0.67 (0.36, 1.26) |  | 0.67 (0.36, 1.25) |  |
|  | Cervical only | 11.63 (1.29, 104.77) |  | 12.43 (1.34, 115.18) |  |
|  | Male only | 0.83 (0.65, 1.06) |  | 0.88 (0.67, 1.17) |  |
|  | Combination known causes | 1.09 (0.78, 1.52) |  | 1.09 (0.77, 1.55) |  |
| Previous unsuccessful IVF (number) | 0 | 1 | 0.35 | 1 | 0.53 |
|  | 1 | 1.29 (0.86, 1.94) |  | 0.57 (0.26, 1.26) |  |
|  | 2 | 1.45 (0.81, 2.60) |  | 0.62 (0.25, 1.51) |  |
|  | >=3 | 1.32 (0.70, 2.49) |  | 0.54 (0.21, 1.37) |  |

**Supplementary material-Table 7: continued**

| Mutually exclusive categories of previous IVF and obstetric history | No previous IVF, 0 pregnancy | 1 | 0.03 | 1 | 0.04 |
| --- | --- | --- | --- | --- | --- |
|  | No previous IVF, at least 1 pregnancy, 0 live births | 1.29 (1.02, 1.63) |  | 1.13 (0.88, 1.45) |  |
|  | No previous IVF, at least 1 pregnancy, at least 1 live birth | 1.09 (0.79, 1.50) |  | 0.97 (0.70, 1.36) |  |
|  | Previous IVF, 0 pregnancy | 1.57 (1.09, 2.25) |  | 2.60 (1.13, 5.93) |  |
|  | Previous IVF, at least 1 pregnancy, 0 live birth | 1.68 (1.09, 2.58) |  | 2.10 (1.18, 3.73) |  |
|  | Previous IVF, at least 1 pregnancy, at least 1 live birth | 0.87 (0.50, 1.49) |  | 0.94 (0.54, 1.64) |  |
| Hormonal preparation | Antioestrogen | 1 | 0.43 | 1 | 0.39 |
|  | Gonadatropin | 1.21 (0.39, 3.81) |  | 1.65 (0.50, 5.38) |  |
|  | Hormone replacement | 0.79 (0.21, 3.02) |  | 1.11 (0.28, 4.40) |  |
| Cycle number | 1 | 1 | 0.97 | 1 | 0.93 |
|  | 2 | 1.00 (0.79, 1.26) |  | 0.99 (0.78, 1.26) |  |
|  | >=3 | 0.97 (0.76, 1.24) |  | 0.95 (0.74, 1.23) |  |
| Source of egg | Donor | 1 | 0.09 | 1 | 0.30 |
|  | Patient | 0.46 (0.19, 1.14) |  | 0.60 (0.23, 1.57) |  |
| Treatment type | IVF | 1 | 0.01 | 1 | 0.24 |
|  | IVF & ICSI | 0.79 (0.66, 0.95) |  | 0.87 (0.69, 1.09) |  |

Cycles include in analyses are those with complete data on all variables and who experienced a singleton birth after IVF. Those with more moderate preterm (i.e. completed gestation weeks between 33-36 weeks) were excluded from analyses so that the comparison is between extreme preterm and term. P-values are likelihood ratio tests of null hypothesis that the odds are the same for each category (i.e. they do not assume linearity)
