# Supplementary material for: Predicting Live Birth, Preterm Delivery, and Low Birth Weight in Infants Born from In Vitro Fertilisation: A Prospective Study of 144,018 Treatment Cycles
Source: PLoS Med. 2011 Jan 4;8(1):e1000386. doi: 10.1371/journal.pmed.1000386 (PMC3014925; doi:10.1371/journal.pmed.1000386)
Supplement: Text S1 — Calculation of predictive probability of live birth after IVF according to Templeton model. (0.03 MB DOC) [file pmed.1000386.s009.doc]

**Text S1: Calculation of predictive probability of live birth after IVF according to Templeton Model**

The predictive probability of achieving a live birth after IVF according to Templeton’s model was calculated as:


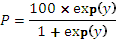


Where y is the linear predictor from the Templeton model and given as:

y = -2.028 + (0.00551 x (*age* - 16)^2^) - (0.00028 x (*age* - 16)^3^) + (
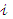
- (0.069 x *number of previous unsuccessful IVF attempts*) - (0.0711 x *tubal reason for infertility*) + (0.7587 x *previous IVF live birth*) + (0.2986 x *IVF pregnancy not resulting in live birth*) + (0.2277 x *previous spontaneous live birth*) + (0.1117 x *previous spontaneous pregnancy not resulting in live birth*)

In the above equation
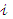
 was a value used to represent duration of infertility and took values:

0.2163 if infertility duration was < 3 years

-0.0839 if infertility duration was 4-6 years

-0.1036 if infertility duration was 7-12 years

-0.4179 if infertility was ≥ 13 years

Tubal infertility, live birth after IVF, previous pregnancy after IVF no live birth, live birth not the result of IVF, previous pregnancy not after IVF and that did not result in live birth, were all binary variables in which values were 1 if the woman had experienced the characteristic and otherwise 0.
