# Supplementary material for: Predicting Live Birth, Preterm Delivery, and Low Birth Weight in Infants Born from In Vitro Fertilisation: A Prospective Study of 144,018 Treatment Cycles
Source: PLoS Med. 2011 Jan 4;8(1):e1000386. doi: 10.1371/journal.pmed.1000386 (PMC3014925; doi:10.1371/journal.pmed.1000386)
Supplement: Text S2 — Equation and tables for derivation of probability of live birth. (0.02 MB DOC) [file pmed.1000386.s010.docx]

**Text S2 - Equation for derivation of probability of live birth**


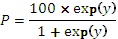


P = probability of live birth per 100 cycles

*y* = -1.1774 + (age and duration effect: Table 1) + (age and source of embryo effect: Table 2) + (ICSI and cause effect: Table 3) + (ICSI and cycle number effect: Table 4) + (previous number of unsuccessful IVF attempts: Table 5) + (previous obstetric history effect: Table 6) + (hormonal preparation effect: Table 7)

**Table 1: Effect to be added for each age and duration of infertility category**

| **Age (years)** | **Duration of infertility (years)** | **Effect** |
| --- | --- | --- |
| 18-34 | <1 | +0.4109 |
|  | 1-3 | +0.1391 |
|  | 4-6 | 0 |
|  | 7-9 | -0.0909 |
|  | 9-12 | -0.1571 |
|  | >12 | -0.1545 |
| 35-37 | <1 | +0.3935 |
|  | 1-3 | +0.0913 |
|  | 4-6 | +0.2000 |
|  | 7-9 | -0.0300 |
|  | 9-12 | -0.1449 |
|  | >12 | -0.1151 |
| 38-39 | <1 | +0.5010 |
|  | 1-3 | 0.0410 |
|  | 4-6 | -0.2586 |
|  | 7-9 | -0.0329 |
|  | 9-12 | -0.0850 |
|  | >12 | -0.0137 |
| 40-42 | <1 | +0.4885 |
|  | 1-3 | +0.0415 |
|  | 4-6 | +0.0513 |
|  | 7-9 | -0.0367 |
|  | 9-12 | -0.0881 |
|  | >12 | -0.1658 |
| 43-44 | <1 | +0.1705 |
|  | 1-3 | -0.1013 |
|  | 4-6 | -0.0336 |
|  | 7-9 | -0.5706 |
|  | 9-12 | -0.8279 |
|  | >12 | -0.4323 |
| 45-50 | <1 | +0.2530 |
|  | 1-3 | -0.0188 |
|  | 4-6 | -0.3816 |
|  | 7-9 | +0.0314 |
|  | 9-12 | +0.0443 |
|  | >12 | -0.4652 |

**Table 2: Effect to be added for each age and source of embryo category**

| **Age** | **Source embryo** | **Effect** |
| --- | --- | --- |
| 18-34 | Donor | 0 |
|  | Patient | +0.0129 |
| 35-37 | Donor | +0.2000 |
|  | Patient | -0.4216 |
| 38-39 | Donor | -0.2586 |
|  | Patient | -0.3436 |
| 40-42 | Donor | +0.0513 |
|  | Patient | -1.2512 |
| 43-44 | Donor | -0.0336 |
|  | Patient | -2.1049 |
| 45-50 | Donor | -0.3816 |
|  | Patient | -2.7981 |

**Table 3: Effect to be added for each ICSI and cause of infertility category**

| **ICSI used** | **Cause of infertility** | **Effect** |
| --- | --- | --- |
| No | Unknown | 0 |
|  | Tubal only | -0.1455 |
|  | Annovulatory only | -0.0763 |
|  | Endometriosis only | -0.0526 |
|  | Cervical only | -1.1661 |
|  | Male only | -0.2728 |
|  | Combined causes | -0.2200 |
| Yes | Unknown | +0.1481 |
|  | Tubal only | -0.8872 |
|  | Annovulatory only | +0.0208 |
|  | Endometriosis only | -0.0499 |
|  | Cervical only | +0.2509 |
|  | Male only | +0.0200 |
|  | Combined causes | -0.0030 |

**Table 4: Effect to be added for each ICSI and treatment cycle number category**

| **ICSI used** | **Treatment cycle number** | **Effect** |
| --- | --- | --- |
| No | 1 | 0 |
|  | 2 | -0.1613 |
|  | >=3 | -0.0368 |
| Yes | 1 | +0.1481 |
|  | 2 | -0.1662 |
|  | >=3 | -0.1927 |

**Table 5: Effect to be added for number of previously unsuccessful IVF attempts**

| **Number of previous unsuccessful IVF attempts** | **Effect** |
| --- | --- |
| 0 | 0 |
| 1 | -0.3210 |
| 2 | -0.3489 |
| 3 | -0.2496 |
| 4 | -0.5931 |
| >=5 | -0.3863 |

**Table 6: Effect to be added for past obstetric history**

| **Past obstetric history** | **Effect** |
| --- | --- |
| No previous IVF, 0 pregnancy | 0 |
| No previous IVF, at least 1 pregnancy, 0 live births | 0.0276 |
| No previous IVF, at least 1 pregnancy, at least 1 live birth | 0.1735 |
| Previous IVF, 0 pregnancy | 0.1280 |
| Previous IVF, at least 1 pregnancy, 0 live birth | 0.0123 |
| Previous IVF, at least 1 pregnancy, at least 1 live birth | 0.4593 |

**Table 7: Effect to be added for hormonal preparation**

| **Hormonal preparation** | **Effect** |
| --- | --- |
| Antioestrogen | 0 |
| Gonadatropin | 0.2900 |
| Hormone replacement | 0.4458 |
